# Association Between Removal of a Warning Against Cephalosporin Use in Patients With Penicillin Allergy and Antibiotic Prescribing

**DOI:** 10.1001/jamanetworkopen.2021.8367

**Published:** 2021-04-29

**Authors:** Eric Macy, Thomas A. McCormick, John L. Adams, William W. Crawford, Myngoc T. Nguyen, Liem Hoang, Victoria Eng, Anna C. Davis, Elizabeth A. McGlynn

**Affiliations:** 1 Department of Allergy and Clinical Immunology, Southern California Permanente Medical Group, San Diego Medical Center, San Diego; 2 Kaiser Permanente Center for Effectiveness & Safety Research, Pasadena, California; 3 Kaiser Permanente Bernard J. Tyson School of Medicine, Pasadena, California; 4 Department of Allergy and Clinical Immunology, Southern California Permanente Medical Group, South Bay Medical Center, Los Angeles; 5 Department of Allergy and Clinical Immunology, The Permanente Medical Group, Oakland Medical Center, Oakland, California; 6 Department of Pharmacy, Arrowhead Regional Medical Center, Colton, California; 7 Department of Allergy and Immunology, Sansum Clinic, Santa Barbara, California; 8 Kaiser Permanente Research, Pasadena, California

## Abstract

**Importance:**

Electronic health records (EHRs) often include default alerts that can influence physician selection of antibiotics, which in turn may be associated with a suboptimal choice of agents and increased antibiotic resistance.

**Objective:**

To examine whether removal of a default alert in the EHR to avoid cephalosporin use in patients with penicillin allergies is associated with changes in cephalosporin dispensing or administration in these patients.

**Design, Setting, and Participants:**

This retrospective cohort study of a natural experiment included data on patients who had received antibiotic treatment in the hospital or outpatient setting in 2 regions of a large, integrated health system in California from January 1, 2017, to December 31, 2018. Of 4 398 792 patients, 4 206 480 met the eligibility criteria: enrollment in the health system during antibiotic use, availability of complete demographic data, and use of antibiotics outside of the washout period.

**Interventions or Exposures:**

Oral or parenteral antibiotics dispensed or administered after removal of an EHR alert to avoid cephalosporin use in patients with a recorded penicillin allergy.

**Main Outcomes and Measures:**

Probability that an antibiotic course was a cephalosporin. A multinomial logistic regression model was used to examine the change in rates of cephalosporin use before and after an EHR penicillin allergy alert was removed in 1 of the study regions. Temporal changes in use rates were controlled for by comparing changes in cephalosporin use among patients with or without a penicillin allergy at the site that removed the warning and among patients at a comparison site that retained the warning. Regression models were used to examine adverse events.

**Results:**

Of the 4 206 480 patients who met all inclusion criteria, 2 465 849 (58.6%) were women; the mean (SD) age was 40.5 (23.2) years. A total of 10 652 014 antibiotic courses were administered or dispensed, divided approximately evenly between the period before and after removal of the warning. Before removal of an alert in the electronic health record system to avoid prescribing of cephalosporins to patients with a penicillin allergy at 1 of the 2 sites, 58 228 courses of cephalosporins (accounting for 17.9% of all antibiotic use at the site) were used among patients with a penicillin allergy; after removal of the alert, administration or dispensing of cephalosporins increased by 47% compared with cephalosporin administration or dispensing among patients without a penicillin allergy at the same site and patients at the comparison site that retained the warning (ratio of ratios of odds ratios [RROR], 1.47; 95% CI, 1.38-1.56) . No significant differences in anaphylaxis (9 total cases), new allergies (RROR, 1.02; 95% CI, 0.93-1.12), or treatment failures (RROR, 1.02; 95% CI, 0.99-1.05) were found at the course level. No significant differences were found in all-cause mortality (ratio of ratios of rate ratios [RRRR], 1.03; 95% CI, 0.94-1.13), hospital days (RRRR, 1.04; 95% CI, 0.99-1.10), and new infections (*Clostridioides difficile*: RRRR, 1.02; 95% CI, 0.84-1.22; methicillin-resistant *Staphylococcus aureus*: RRRR, 0.87; 95% CI, 0.75-1.00; and vancomycin-resistant *Enterococcus*: RRRR, 0.82; 95% CI, 0.55-1.22) at the patient level.

**Conclusions and Relevance:**

In this cohort study, removal of a warning in the electronic health record to avoid cephalosporin use in patients with penicillin allergies was associated with increased administration and dispensing of cephalosporin. This simple and rapidly implementable system-level intervention may be useful for improvement in antibiotic stewardship.

## Key Points

**Question:**

Is removal of a warning to avoid cephalosporin use in patients with penicillin allergies associated with an increase in cephalosporin dispensing or administration?

**Findings:**

In this cohort study of 4 398 792 patients who had received antibiotic treatment, after an alert in the electronic health record system to avoid prescribing of cephalosporins to patients with a penicillin allergy was removed at 1 of 2 health system sites, cephalosporin dispensing or administration increased significantly among patients with a penicillin allergy at that site compared with patients without a penicillin allergy at the same site and patients at the comparison site that retained the warning.

**Meaning:**

In this study, removal of a warning in the electronic health record to avoid cephalosporin use in patients with penicillin allergies was associated with increased dispensing and administration of cephalosporin.

## Teaser

This cohort study assesses whether removal of a warning against use of cephalosporins in the electronic health record (EHR) of patients with penicillin allergy was associated with changes in the dispensing or administration of cephalosporins.

## Introduction

Increases in drug-resistant organisms are a global threat, and thus antibiotic stewardship is a critical priority.^1^ Approaches to antibiotic stewardship include both reducing unnecessary use of antibiotics and choosing the most appropriate agents. β-Lactams are the preferred treatment for many common pathogens, such as group A and B *Streptococcus* species, *Neisseria gonorrhoeae*, *Neisseria meningitidis*, and *Staphylococcus aureus*.^2^ β-Lactams are also the preferred prophylactic agents for dental procedures and many other surgical procedures.^3^ Physicians may, however, choose a second-line antibiotic in the presence of drug allergies or potential cross-reactivity with β-lactams.^4,5,6^

Point-of-care alerts to inform prescribing decisions by physicians and to enhance patient safety have been among the reasons for use of electronic health records (EHRs). In response to Meaningful Use requirements issued by the Office of the National Coordinator for Health Information Technology, many EHRs include an imbedded function to warn against prescribing cephalosporins to patients with a penicillin allergy.^7,8,9^ This warning is not consistent with current evidence.^2^ For example, no clinically significant immunologic cross-reactivity between penicillin and cephalosporins has been shown.^10,11^ Furthermore, avoiding β-lactams when they are the drug of choice is associated with worse outcomes, such as increased risk of surgical site infections,^5,12,13^ nephrotoxicity,^14^ and *Clostridioides difficile* infection.^15^ In addition, use of second-line antibiotic classes in patients with penicillin allergies may be associated with other adverse effects, decreased safety, and antimicrobial resistance.^4,6,16^

In December 2017, after a review of the literature and clinical deliberations among leaders in infectious diseases in the Southern California Permanente Medical Group, Kaiser Permanente Southern California removed a warning in its EHR to avoid prescribing cephalosporins to patients with a penicillin allergy. In the current study, we examined the association of this change with choice of antibiotic, incidence of antibiotic allergy, incidence of cephalosporin-associated anaphylaxis, and rate of penicillin allergy–associated morbidities in patients with a penicillin allergy.

## Methods

This retrospective cohort study of a natural experiment included members enrolled in either Kaiser Permanente Southern California or Kaiser Permanente Northern California health plans who received antibiotic treatment between January 1, 2017, and December 31, 2018. Kaiser Permanente Southern California, which removed the alert, was the intervention site, and Kaiser Permanente Northern California, which did not remove the EHR alert, was the comparison site for the natural experiment. The institutional review boards of both sites approved this study and waived the requirement for informed consent because the research involved no more than minimal risk to participants, the waiver would not adversely affect participants’ rights and welfare, the research could not practically be carried out without the waiver, and participants were provided with additional pertinent information after participation. The study followed the Strengthening the Reporting of Observational Studies in Epidemiology (STROBE) reporting guideline.^17^ Additional details are provided in the eMethods in the Supplement.

At each site, a difference-in-differences^18^ design comparing changes in the cohort of patients allergic to penicillin with changes in the cohort of patients without penicillin allergy was used. Our focus was on understanding the changes in prescribing patterns and monitoring for adverse effects after removal of the EHR alert. Together, the health plans cover approximately 9 million members who receive care at 36 hospitals and 491 outpatient facilities from more than 17 000 physicians. The enrolled populations are broadly representative of the general population in California.^19,20^

### Data Sources

Data were extracted from existing health plan and care delivery sources, including membership records, integrated claims, ambulatory and hospital-based EHRs, and other administrative data. We calculated baseline patient characteristics using data starting from January 1, 2016, and extracted outcome data through January 31, 2019, to allow for follow-up time to measure outcomes for courses of antibiotics started through December 31, 2018.

### Study Definitions

We included all oral and parenteral antibiotic treatment courses dispensed or administered to enrolled members in the ambulatory or hospital setting for 12 antibiotic categories: penicillin, cephalosporin, other β-lactams, clindamycin, macrolides, metronidazole, nitrofurantoin, quinolones, sulfonamides, tetracyclines, vancomycin, and other antibiotics (a detailed list is provided in eTable 1 in the Supplement). Antibiotic use that occurred outside membership periods was excluded. We combined periods of exposure into the same course of antibiotic use if they involved the same drug by the same route and were separated by less than 2 days. The earliest date that the antibiotic was dispensed or administered was used to assign each course to the period before (January 1, 2017-December 12, 2017) or after (December 28, 2017-December 31, 2018) the change in the alert on December 20, 2017; we excluded data on antibiotics dispensed or administered in the 7 days before and 7 days after the change in the alert. The patient’s penicillin allergy status was ascertained on the day before the start date of each antibiotic course.

We used any entry in the EHR allergy field to define the presence of a drug allergy. We did not require confirmed IgE-mediated or T-cell–mediated hypersensitivity. Although misclassification of penicillin allergy status in the EHR can occur,^21^ we used the presence of an allergy record in the EHR to define *allergy* because this record triggers the automated warning that was being studied. We extracted complete histories of antibiotic allergies to identify periods of allergies to antibiotics in the 12 categories during the study. New antibiotic allergies were defined as new allergy records after use of an antibiotic in the same category within 30 days of the start of the treatment course.

Potential cases of cephalosporin-associated anaphylaxis in patients with a penicillin allergy were identified with *International Statistical Classification of Diseases and Related Health Problems, Tenth Revision (ICD-10)* codes T78.2 or T88.6 recorded on the same day that a course of parenteral cephalosporins was started or within 1 day of starting a course of oral cephalosporins. Each potential case was manually audited by chart review (E.M. and W.W.C. for the intervention site and M.T.N. for the comparison site) to determine whether it met the current diagnostic criteria for antibiotic-associated anaphylaxis.^22^ As a proxy for antibiotic treatment failure, we used the start of a new course of antibiotics in a different category within 30 days of the start of a course of monotherapy.^16^

The rate of days spent in the hospital (hereafter *hospital days*) per person-year was defined as the number of days in the period that the patient spent in the hospital (including time contributed by the emergency department and observation days that were linked to an inpatient admission) divided by the total member-days in the period. The rate of new infections per person-year was defined as patients having 1 or more new *C difficile*, methicillin-resistant *S aureus* (MRSA), or vancomycin-resistant *Enterococcus* (VRE) infections during the period. We selected these infections a priori based on prior studies^4,23,24^ and clinical input. Infections were identified based on positive laboratory results or diagnoses from encounters in the hospital or outpatient setting. *ICD-10* diagnosis codes starting with A04.7 were used to identify *C difficile* infection; A41.02, A49.02, B95.62, or J15.212 were used to identify MRSA infection; and Z16.21 and Z16.22 were used to identify VRE infection. Infections were considered new if there were no indications of VRE or MRSA infection in the prior 30 days or *C difficile* infection in the prior 90 days.

We defined comorbidities using the diagnoses included in the Charlson Comorbidity Index.^25^ We defined hospital encounters for surgery or labor and delivery using diagnosis-related group codes.

### Statistical Analysis

The primary outcome was the category of antibiotic use measured by the change in the probability of cephalosporin use among patients with a penicillin allergy at the intervention site after the removal of the warning. We used a multinomial logistic regression model with the 12 antibiotic categories as the outcome. Generalized estimating equation–based adjustments to the SEs were performed using an independent working correlation matrix (details are provided in the eMethods in the Supplement). For each course of antibiotic treatment, the model included an indicator for site, period (before or after removal of the alert), and presence or absence of a penicillin allergy. The model included these main effects, as well as all 2-way interactions and the 3-way interaction. Patient sex, age, and self-reported race/ethnicity and the age-sex interaction were included as covariates in the model to adjust for differences between the sites.

This model simultaneously controlled for temporal effects using 2 comparison populations. First, we examined the change in cephalosporin prescriptions among patients without penicillin allergies, which should have been unaffected by the removal of the warning. This was an adjusted ratio of odds ratios (RORs) calculation. Second, we examined the equivalent RORs for the comparison site to further control for temporal changes in antibiotic use. The outcome was formulated as a ratio of these 2 RORs (RROR) and was modeled with multinomial logistic regression. The regression coefficient for the 3-way interaction represents the change in antibiotic prescribing as the natural log of the RROR. We fit models for all courses and fit separate models for oral and parenteral courses.

We calculated several secondary outcomes to explore whether removal of the warning was associated with patient outcomes. At the treatment-course level, we examined anaphylaxis, new antibiotic allergies, and antibiotic treatment failure. At the patient level, we assessed all-cause mortality, hospital days, and new infections per person-year. Regression models for the secondary outcomes used the same independent variables and interactions as in the model for the primary outcome. The details of the models for secondary outcomes varied, but all were formulated using the ROR approach described for the primary outcome. Course-level outcomes were RRORs, and patient-level outcomes were ratios of ratios of rate ratios (RRRRs). Dimensions of the differences included the model distributions and link functions, criteria for including courses and person-time, consideration of different cephalosporin generations, and membership criteria. More details are given in the eMethods and eTable 3 in the Supplement.

In addition, we fit separate models for the secondary outcomes that included the penicillin allergy indicator only as a main effect but not in interactions with other independent variables. These models estimated the extent to which patients with a penicillin allergy had a higher rate of the adverse outcome than patients without a penicillin allergy.

We tested the sensitivity of the results for the primary outcome after adding covariates to the models, including indicators for allergies to other antibiotic categories, the diagnosis group associated with the course, and the presence of individual comorbidities at the start of the course and indicators that the course occurred during a hospitalization for surgery or labor and delivery. We tested how the results changed when the main variables interacted with the other covariates as well as after adding effect modification of the 3-way interaction by adding the 4-way interactions with age, sex, and race/ethnicity. In addition, we tested whether the results were sensitive to different variable definitions by assigning the penicillin allergy on the date when the treatment was first dispensed or administered (rather than the day before) and including only each patient’s first or last course in the model. Analyses were conducted using SAS, version 7.1 (SAS Institute).

## Results

We identified 4 398 792 unique patients with antibiotic use during the study period. Of these, 143 606 (3.3%) were excluded because they were not enrolled at the time of their antibiotic use; 281 (0.01%) were excluded owing to missing or inconsistent birth date, sex, or death date; and 48 425 (1.1%) were excluded because their antibiotic use occurred during the washout period. Thus, 4 206 480 patients (2 252 525 at the intervention site and 1 953 955 at the comparison site) were included in the analysis: 2 465 849 (58.6%) were women, 1 827 714 (43.4%) were non-Hispanic White, and the mean (SD) age was 40.5 (23.2) years at the start of the study period. Patient demographics differed between the 2 sites; members at the intervention site were younger, more likely to be Black or Hispanic, and less likely to be Asian or White (Table 1). The crude mortality rate at the comparison site was higher than that at the intervention site (1.8% [34 485 deaths] vs 1.5% [32 734 deaths]), which is consistent with previously published studies.^26,27^ Patients contributed a mean (SD) of 2.5 (2.7) treatment courses to the study, and 9.4% of patients had a penicillin allergy at the start of the study period.

**Table 1.** Characteristics of the 4 206 480 Patients Included in the Study

Characteristic	Patients, No. (%)	
	Intervention site (n = 2 252 525)	Comparison site (n = 1 953 955)
Sex		
Female	1 320 984 (58.6)	1 144 865 (58.6)
Male	931 541 (41.4)	809 090 (41.4)
Age at study start, y		
Mean (SD)	39.9 (23.1)	41.2 (23.2)
Median (IQR)	40 (22-58)	41 (23-59)
Race/ethnicity		
American Indian or Alaska Native (non-Hispanic)	23 944 (1.1)	25 883 (1.3)
Asian or Pacific Islander (non-Hispanic)	216 343 (9.6)	337 055 (17.3)
Black (non-Hispanic)	203 047 (9.0)	149 368 (7.6)
Hispanic	801 339 (35.6)	342 225 (17.5)
White (non-Hispanic)	847 734 (37.6)	979 980 (50.2)
Other race/ethnicity^a^	160 118 (7.1)	119 444 (6.1)
Penicillin allergy at study start	201 293 (8.9)	192 534 (9.9)
Died during study	32 734 (1.5)	34 485 (1.8)
Continuously enrolled during study	1 839 686 (81.7)	1 615 251 (82.7)
≥1 Oral course of antibiotic treatment	2 144 346 (95.2)	1 835 996 (94.0)
≥1 Parenteral course of antibiotic treatment	502 280 (22.3)	437 959 (22.4)
Antibiotic treatment courses, No.		
Mean (SD)	2.6 (2.7)	2.5 (2.6)
Median (IQR)	2 (1-3)	2 (1-3)
Received antibiotic treatment in the period before warning removal	1 420 320 (63.1)	1 216 371 (62.3)
Received antibiotic treatment in the period after warning removal	1 496 480 (66.4)	1 274 700 (65.2)

Abbreviation: IQR, interquartile range.

^a^ Other race/ethnicity indicates not specified, unknown, or patient declined to state.

We included 10 652 014 antibiotic courses in the analysis (Table 2); 51.8% were in the period after removal of the alert, 18.0% were parenteral courses, and 12.1% were dispensed or administered to patients with a documented penicillin allergy (eTable 4 in the Supplement). Cephalosporin use (as a fraction of all antibiotic use) increased among patients with a penicillin allergy at the intervention site from 17.9% of courses in the period before removal of the alert to 27.0% of courses in the period after removal of the alert (Table 2). As cephalosporin use increased, use of some categories of antibiotics decreased in the period after removal of the alert. For example, among patients with penicillin allergy at the intervention site, use of clindamycin decreased from 13.7% to 11.4% and use of quinolones decreased from 12.8% to 10.5%. The same general pattern was present for oral and parenteral courses (eTable 5 in the Supplement). Most cephalosporin courses (71.7%) were first generation (eTables 2 and 6 in the Supplement), and the increase in use was mainly in this generation (eTable 7 in the Supplement). The change in rates of use of cephalosporin, clindamycin, or quinolones at the comparison site before vs after removal of the alert was smaller; among patients with penicillin allergy, cephalosporin use increased from 15.3% to 16.2%, clindamycin use increased from 14.0% to 14.2%, and quinolone use decreased from 14.7% to 14.2%.

**Table 2.** Patterns of Antibiotic Use by Antibiotic Category, Study Period, Penicillin Allergy Status, and Study Site

Antibiotic category	Antibiotic treatment courses dispensed or administered, %							
	Intervention site				Comparison site			
	No allergy		Allergy		No allergy		Allergy	
	Before^a^	After^**b**^	Before^a^	After^**b**^	Before^a^	After^**b**^	Before^a^	After^**b**^
Antibiotic treatment courses, No.	2 480 535	2 679 107	324 856	349 847	2 029 273	2 174 367	297 710	316 319
Penicillin	29.5	29.5	2.1	2.2	29.8	29.5	2.0	2.1
Cephalosporin	25.8	26.4	17.9	27.0	23.7	24.4	15.3	16.2
Other β-lactams	0.2	0.2	0.5	0.4	0.2	0.2	1.0	0.8
Clindamycin	2.6	2.5	13.7	11.4	2.2	2.2	14.0	14.2
Macrolides	12.5	12.2	19.9	18.3	13.6	12.8	19.0	17.4
Metronidazole	4.8	4.9	5.4	5.5	4.1	4.5	5.1	5.1
Nitrofurantoin	2.5	2.8	4.3	3.9	3.1	3.4	4.2	4.6
Quinolones	7.9	7.3	12.8	10.5	7.8	7.3	14.7	14.2
Sulfonamides	4.6	4.3	7.4	5.8	4.5	4.2	7.0	6.7
Tetracyclines	7.2	7.3	11.4	11.4	8.0	8.5	12.7	13.9
Vancomycin	1.2	1.3	2.6	2.0	1.5	1.5	2.9	2.9
Other antibiotics	1.2	1.2	1.8	1.5	1.6	1.5	2.1	2.1

^a^ One year before removal of the penicillin allergy warning from the electronic health record.

^b^ One year after removal of the penicillin allergy warning from the electronic health record.

We found a significant increase of 47% in cephalosporin use among patients with penicillin allergy at the intervention site compared with patients in the other groups (RROR, 1.47; 95% CI, 1.38-1.56). The RRORs for the 12 antibiotic categories are shown in the Figure and in eTables 8-10 in the Supplement. The RRORs for other categories generally decreased or stayed the same in the period after removal of the alert. The exception was metronidazole (RROR, 1.10; 95% CI, 1.03-1.18), but this result appeared to be an artifact of ratios of small numbers (Table 2). The change in oral cephalosporin use was similar to the main result calculated for all courses, with an RROR of 1.51 (95% CI, 1.41-1.61) (eTable 9 in the Supplement). The crude increase in the rate of prescribing of parenteral cephalosporins (from 35.5% to 54.3%) among patients with penicillin allergy at the intervention site was larger than that for oral cephalosporins (from 14.2% to 21.1%), but the RROR was smaller (1.29; 95% CI, 1.10-1.51) because of a concurrent increase in use of parenteral cephalosporins at the comparison site.

**Figure.**
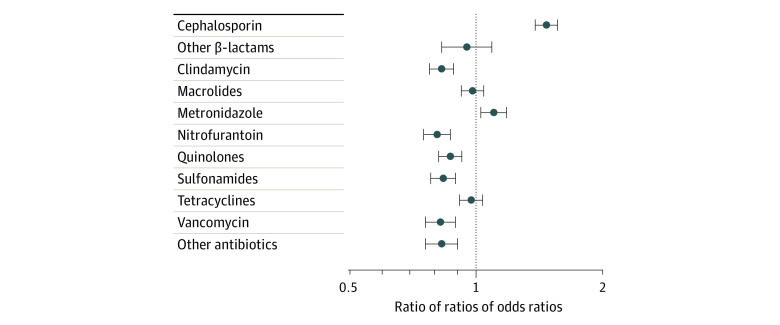
Multinomial Logistic Regression of Changes in Antibiotic Use Among Patients With Penicillin Allergies Odds ratio of a patient with penicillin allergy receiving a course of antibiotic treatment, by category, at the intervention site before vs after removal of a warning against cephalosporin use from the electronic health record compared with odds ratios of patients without a penicillin allergy and those at the comparison site receiving the treatment in those periods. Whiskers indicate 95% CIs; vertical line represents a ratio of ratios of rate ratios of 1.0, indicating no change in antibiotic use (compared with other groups) after the removal of the warning.

The difference in the rates of anaphylaxis among individuals with penicillin allergies who used cephalosporin between the sites was not significant. The results for the other course-level outcome calculations are summarized in Table 3. New allergies and antibiotic treatment failure were more likely to occur among patients with a penicillin allergy than among those without a penicillin allergy (eTables 11-15 in the Supplement). The RRORs for these outcomes were not statistically significant when calculated with all courses (new allergies, 1.02 [95% CI, 0.93-1.12]; treatment failure, 1.02 [95% CI, 0.99-1.05]) or when calculated separately including only oral courses (new allergies, 1.00 [95% CI, 0.91-1.10]; treatment failure, 1.02 [95% CI, 0.99-1.06]) or parenteral courses (new allergies, 1.23 [95% CI, 0.94-1.61]; treatment failure, 0.96 [95% CI, 0.90-1.03]). Furthermore, patients with a penicillin allergy at the intervention site who used a cephalosporin had a similar rate of new cephalosporin allergies within 30 days during the 2 periods (1.0% of courses in the period before removal of the alert and 0.9% of courses in the period after removal of the alert) (eTable 11 in the Supplement).

**Table 3.** Antibiotic Treatment Course–Level Outcome Results for All Patients and Patients With vs Without Penicillin Allergies

Outcome	All courses	Oral courses	Parenteral courses
New antibiotic allergy within 30 d			
Crude RROR	1.02	1.00	1.23
RROR from binomial logistic regression model (95% CI)	1.02 (0.93-1.12)	1.00 (0.91-1.10)	1.23 (0.94-1.61)
Overall OR for patients with vs without penicillin allergy (95% CI)	1.62 (1.58-1.66)	1.58 (1.54-1.62)	1.91 (1.79-2.05)
Treatment failure within 30 d			
Crude RROR	1.02	1.02	0.96
RROR from binomial logistic regression model (95% CI)	1.02 (0.99-1.05)	1.02 (0.99-1.06)	0.96 (0.90-1.03)
Overall OR for patients with vs without penicillin allergy (95% CI)	1.10 (1.10-1.11)	1.10 (1.10-1.11)	1.18 (1.16-1.21)

Abbreviations: OR, odds ratio; RROR, ratio of ratios of odds ratios.

The RRRR was not significantly different from 1 for all-cause mortality (1.03; 95% CI, 0.94-1.13), hospital days (1.04; 95% CI, 0.99-1.10), or new *C difficile* (1.02; 95% CI, 0.84-1.22), MRSA (0.87; 95% CI, 0.75-1.00), or VRE (0.82; 95% CI, 0.55-1.22) infection among all patients (Table 4). The RRRRs were also not significantly different when the hospital day and new infection calculations were limited to only patients who received parenteral courses. Patients with penicillin allergies had worse outcomes, and the outcomes generally improved after removal of the alert in each patient group (eTables 16-19 in the Supplement).

**Table 4.** Person-Level Outcome Results for All Patients and Patients With vs Without Penicillin Allergies

Outcomes	All patients	Patients receiving parenteral treatment
All-cause mortality		
Crude RRRR	1.02	NA
RRRRs from Poisson regression model (95% CI)^a^	1.03 (0.94-1.13)	NA
Overall rate for patients with vs without penicillin allergy (95% CI)	1.03 (1.01-1.06)	NA
Hospital days		
Crude RRRR	1.04	1.05
RRRR from Poisson regression model (95% CI)	1.04 (0.99-1.10)	1.05 (0.99-1.11)
Overall rate for patients with vs without penicillin allergy (95% CI)	1.09 (1.08-1.11)	1.12 (1.10-1.14)
New *Clostridioides difficile* infection		
Crude RRRR	1.02	1.01
RRRR from Poisson regression model (95% CI)	1.02 (0.84-1.22)	1.02 (0.82-1.26)
Overall rate for patients with vs without penicillin allergy (95% CI)	1.23 (1.17-1.29)	1.17 (1.11-1.24)
New MRSA infection		
Crude RRRR	0.87	0.88
RRRR from Poisson regression model (95% CI)	0.87 (0.75-1.00)	0.88 (0.73-1.05)
Overall rate for patients with vs without penicillin allergy (95% CI)	1.06 (1.02-1.10)	1.14 (1.09-1.20)
New VRE infection		
Crude RRRR	0.82	0.77
RRRR from Poisson regression model (95% CI)	0.82 (0.55-1.22)	0.78 (0.51-1.18)
Overall rate for patients with vs without penicillin allergy (95% CI)	1.39 (1.26-1.53)	1.38 (1.24-1.53)

Abbreviations: MRSA, methicillin-resistant *Staphylococcus aureus*; NA, not applicable; RRRR, ratio of ratios of rate ratios; VRE, vancomycin-resistant *Enterococcus*.

^a^ Details are provided in the eMethods in the Supplement.

The results were robust to several sensitivity analyses (eTable 20 in the Supplement). The effect size was generally larger in the more complicated models, although the 95% CIs overlapped with the 95% CIs from the main model (eTable 20 in the Supplement). In models that included all courses, the value of the RROR of interest ranged from 1.46 to 1.64. For models that included only oral or only parenteral courses, the RROR values ranged from 1.52 to 1.71 and 1.20 to 1.37, respectively (eTable 20 in the Supplement). The model with effect modification by age, sex, and race/ethnicity suggested that the change in the warning was associated with fewer changes in antibiotic use patterns for patients younger than 18 years (RROR, 0.7; 95% CI, 0.6-0.9).

## Discussion

In this retrospective cohort study of a natural experiment, cephalosporin use increased from 17.9% to 27.0% among patients at the intervention site, where the warning not to prescribe cephalosporins to patients with a penicillin allergy was removed, compared with a change from 15.3% to 16.2% at the comparison site, which retained the warning. This translated to a statistically significant increase in cephalosporin use at the intervention site (RROR, 1.47; 95% CI, 1.38-1.56) during the period after removal of the penicillin allergy warning. We found no significant differences in the rates of anaphylaxis, allergies to new antibiotic classes, antibiotic treatment failure, all-cause mortality, hospital days, and new infections after the change.

Although the rate of adverse outcomes did not increase after removal of the warning, patients with penicillin allergy experienced worse outcomes in general than did those with no penicillin allergy. These results are consistent with prior studies^16,28^ suggesting higher rates of new antibiotic allergies among patients with a penicillin allergy who receive any antibiotic compared with patients without a penicillin allergy. The results are also consistent with prior studies showing higher rates of infection^16,23,28^ and mortality^16,29^ among patients with a penicillin allergy.

Removal of the penicillin allergy warning in the EHR represents a simple and rapidly implementable system-level intervention to potentially improve antibiotic stewardship. In addition, removal of an unsupported warning in the EHR may be associated with reduced alert fatigue among clinicians.^7^ This study’s findings may encourage other health care systems to remove the warning, which may improve antibiotic stewardship.

### Limitations

This study has limitations. Because it was a retrospective study, we cannot rule out that unmeasured confounders may explain some of the associations. We attempted to control for this by using a before-and-after design and making comparisons with changes in the 2 patient groups that were unaffected by the removal of the warning (those at the comparison site, where the warning was not changed, and those at the intervention site who did not have a penicillin allergy, to whom the warning did not apply). Although we adjusted the results for basic confounders, it is possible that unmeasured patient characteristics were contributing biases. In addition, although none of the changes in the secondary outcomes were statistically significant, the 95% CIs were generally large enough to include at least a 10% increase or decrease in each outcome. Thus, we cannot rule out the possibility that removing the warning was associated with changes in patient outcomes.

## Conclusions

In this cohort study, removal of a warning in the EHR to avoid cephalosporin use in patients with penicillin allergies was associated with increased prescribing of cephalosporins and decreased prescribing of some second-line antibiotics. Overall rates of adverse antibiotic-associated reactions or specific serious cephalosporin-associated morbidities were similar before and after removal of the warning. Removing the warning was not associated with a reduction in the known morbidities associated with an unconfirmed penicillin allergy. Greater attention should be given to the accuracy with which patients are labeled as having a penicillin allergy.^30^

## Notes

Supplement. **eMethods.** Additional methodology details **eTable 1.** Antibiotic names and categories **eTable 2.** Classification of cephalosporin antibiotics by generation **eTable 3.** Definitions and modeling strategies for secondary outcomes **eTable 4.** Descriptive statistics for courses in the analysis **eTable 5.** Patterns of antibiotic use, by category, before and after warning was removed in the intervention site, by penicillin allergy status and region, for oral and parenteral courses separately **eTable 6.** Generation of cephalosporin courses in the analysis **eTable 7.** Change in cephalosporin use by generation (all courses) **eTable 8.** Ratios of ratios of odds ratios for changes in antibiotic use for patients with penicillin allergies **eTable 9.** Ratios of ratios of odds ratios for changes in oral antibiotic use for patients with penicillin allergies **eTable 10.** Ratios of ratios of odds ratios for changes in parenteral antibiotic use for patients with penicillin allergies **eTable 11.** Fraction of courses with a new allergy within 30 days **eTable 12.** Fraction of oral and parenteral courses with a new allergy within 30 days **eTable 13.** Odds ratios of new allergy within 30 days **eTable 14.** Crude odds ratios of antibiotic treatment failure within 30 days of a course of monotherapy **eTable 15.** Odds ratios of antibiotic treatment failure within 30 days of a course of monotherapy **eTable 16.** Crude and standardized mortality rates by penicillin allergy status and region **eTable 17.** Crude rate of hospital days per person year **eTable 18.** Crude rates of new infections per 1,000 person years **eTable 19.** Crude rates of new infections for patients with parenteral courses of antibiotics **eTable 20.** Ratios of ratios of odds ratios for change in cephalosporin use for patients with penicillin allergies from sensitivity analyses

## References

[1] World Health Organization. Prioritization of Pathogens to Guide Discovery, Research and Development of New Antibiotics for Drug-Resistant Bacterial Infections, Including Tuberculosis. World Health Organization, 2017.

[2] Shenoy ES, Macy E, Rowe T, Blumenthal KG. Evaluation and management of penicillin allergy: a review. JAMA. 2019;321(2):188-199. doi:10.1001/jama.2018.19283 30644987

[3] Bratzler DW, Dellinger EP, Olsen KM, ; American Society of Health-System Pharmacists (ASHP); Infectious Diseases Society of America (IDSA); Surgical Infection Society (SIS); Society for Healthcare Epidemiology of America (SHEA). Clinical practice guidelines for antimicrobial prophylaxis in surgery. Surg Infect (Larchmt). 2013;14(1):73-156. doi:10.1089/sur.2013.9999 23461695

[4] Jeffres MN, Narayanan PP, Shuster JE, Schramm GE. Consequences of avoiding β-lactams in patients with β-lactam allergies. J Allergy Clin Immunol. 2016;137(4):1148-1153. doi:10.1016/j.jaci.2015.10.026 26688516

[5] Blumenthal KG, Ryan EE, Li Y, Lee H, Kuhlen JL, Shenoy ES. The impact of a reported penicillin allergy on surgical site infection risk. Clin Infect Dis. 2018;66(3):329-336. doi:10.1093/cid/cix794 29361015 PMC5850334

[6] Chiriac AM, Banerji A, Gruchalla RS, . Controversies in drug allergy: drug allergy pathways. J Allergy Clin Immunol Pract. 2019;7(1):46-60.e4. doi:10.1016/j.jaip.2018.07.037 30573422 PMC6466632

[7] McCoy AB, Thomas EJ, Krousel-Wood M, Sittig DF. Clinical decision support alert appropriateness: a review and proposal for improvement. Ochsner J. 2014;14(2):195-202. 24940129 PMC4052586

[8] Slight SP, Berner ES, Galanter W, . Meaningful use of electronic health records: experiences from the field and future opportunities. JMIR Med Inform. 2015;3(3):e30. doi:10.2196/medinform.4457 26385598 PMC4704893

[9] Kuperman GJ, Bobb A, Payne TH, . Medication-related clinical decision support in computerized provider order entry systems: a review. J Am Med Inform Assoc. 2007;14(1):29-40. doi:10.1197/jamia.M2170 17068355 PMC2215064

[10] Saxon A, Beall GN, Rohr AS, Adelman DC. Immediate hypersensitivity reactions to beta-lactam antibiotics. Ann Intern Med. 1987;107(2):204-215. doi:10.7326/0003-4819-107-2-204 3300459

[11] Daulat S, Solensky R, Earl HS, Casey W, Gruchalla RS. Safety of cephalosporin administration to patients with histories of penicillin allergy. J Allergy Clin Immunol. 2004;113(6):1220-1222. doi:10.1016/j.jaci.2004.03.023 15214363

[12] Thayer A, Smith K, Clark D, Cefazolin-based antimicrobial prophylaxis may reduce surgical site infections in patients undergoing peripheral vascular bypass surgery. Open Forum Infect Dis. 2016;3(suppl 1):1467. doi:10.1093/ofid/ofw172.1169

[13] Hawn MT, Richman JS, Vick CC, . Timing of surgical antibiotic prophylaxis and the risk of surgical site infection. JAMA Surg. 2013;148(7):649-657. doi:10.1001/jamasurg.2013.134 23552769

[14] Tice AD, Rehm SJ, Dalovisio JR, ; IDSA. Practice guidelines for outpatient parenteral antimicrobial therapy. IDSA guidelines. Clin Infect Dis. 2004;38(12):1651-1672. doi:10.1086/420939 15227610

[15] Baxter R, Ray GT, Fireman BH. Case-control study of antibiotic use and subsequent *Clostridium difficile*–associated diarrhea in hospitalized patients. Infect Control Hosp Epidemiol. 2008;29(1):44-50. doi:10.1086/524320 18171186

[16] West RM, Smith CJ, Pavitt SH, . “Warning: allergic to penicillin”: association between penicillin allergy status in 2.3 million NHS general practice electronic health records, antibiotic prescribing and health outcomes. J Antimicrob Chemother. 2019;74(7):2075-2082. doi:10.1093/jac/dkz127 31225607

[17] von Elm E, Altman DG, Egger M, Pocock SJ, Gøtzsche PC, Vandenbroucke JP; STROBE Initiative. The Strengthening the Reporting of Observational Studies in Epidemiology (STROBE) statement: guidelines for reporting observational studies. J Clin Epidemiol. 2008;61(4):344-349. doi:10.1016/j.jclinepi.2007.11.008 18313558

[18] Dimick JB, Ryan AM. Methods for evaluating changes in health care policy: the difference-in-differences approach. JAMA. 2014;312(22):2401-2402. doi:10.1001/jama.2014.16153 25490331

[19] Koebnick C, Langer-Gould AM, Gould MK, . Sociodemographic characteristics of members of a large, integrated health care system: comparison with US Census Bureau data. Perm J. 2012;16(3):37-41. doi:10.7812/TPP/12-031 23012597 PMC3442759

[20] Gordon N, Lin T. The Kaiser Permanente Northern California adult member health survey. Perm J. 2016;20(4):15-225. doi:10.7812/TPP/15-225 27548806 PMC5101088

[21] Trubiano JA, Adkinson NF, Phillips EJ. Penicillin allergy is not necessarily forever. JAMA. 2017;318(1):82-83. doi:10.1001/jama.2017.6510 28672303 PMC5935455

[22] Macy E, Contreras R. Adverse reactions associated with oral and parenteral use of cephalosporins: a retrospective population-based analysis. J Allergy Clin Immunol. 2015;135(3):745-52.e5. doi:10.1016/j.jaci.2014.07.062 25262461

[23] Macy E, Contreras R. Health care use and serious infection prevalence associated with penicillin “allergy” in hospitalized patients: a cohort study. J Allergy Clin Immunol. 2014;133(3):790-796. doi:10.1016/j.jaci.2013.09.021 24188976

[24] Blumenthal KG, Lu N, Zhang Y, Li Y, Walensky RP, Choi HK. Risk of meticillin resistant *Staphylococcus aureus* and *Clostridium difficile* in patients with a documented penicillin allergy: population based matched cohort study. BMJ. 2018;361:k2400. doi:10.1136/bmj.k2400 29950489 PMC6019853

[25] Quan H, Sundararajan V, Halfon P, . Coding algorithms for defining comorbidities in *ICD-9-CM* and *ICD-10* administrative data. Med Care. 2005;43(11):1130-1139. doi:10.1097/01.mlr.0000182534.19832.83 16224307

[26] Sidney S, Sorel ME, Quesenberry CP, . Comparative trends in heart disease, stroke, and all-cause mortality in the United States and a large integrated healthcare delivery system. Am J Med. 2018;131(7):829-836.e1. doi:10.1016/j.amjmed.2018.02.014 29625083 PMC6005733

[27] Chen W, Yao J, Liang Z, . Temporal trends in mortality rates among Kaiser Permanente Southern California health plan enrollees, 2001-2016. Perm J. 2019;23:18-213. 31050639 10.7812/TPP/18-213 PMC6499114

[28] Macy E, Ho NJ. Multiple drug intolerance syndrome: prevalence, clinical characteristics, and management. Ann Allergy Asthma Immunol. 2012;108(2):88-93. doi:10.1016/j.anai.2011.11.006 22289726

[29] Blumenthal KG, Peter JG, Trubiano JA, Phillips EJ. Antibiotic allergy. Lancet. 2019;393(10167):183-198. doi:10.1016/S0140-6736(18)32218-9 30558872 PMC6563335

[30] Macy E, Shu YH. The effect of penicillin allergy testing on future healthcare utilization: a matched cohort study. J Allergy Clin Immunol Pract. 2017;5(3):705-710. doi:10.1016/j.jaip.2017.02.012 28366717

