# Supplementary material for: Association Between Removal of a Warning Against Cephalosporin Use in Patients With Penicillin Allergy and Antibiotic Prescribing
Source: JAMA Netw Open. 2021 Apr 29;4(4):e218367. doi: 10.1001/jamanetworkopen.2021.8367 (PMC8085727; doi:10.1001/jamanetworkopen.2021.8367)
Supplement: Supplement. — eMethods. Additional methodology details eTable 1. Antibiotic names and categories eTable 2. Classification of cephalosporin antibiotics by generation eTable 3. Definitions and modeling strategies for secondary outcomes eTable 4. Descriptive statistics for courses in the analysis eTable 5. Patterns of antibiotic use, by category, before and after warning was removed in the intervention site, by penicillin allergy status and region, for oral and parenteral courses separately eTable 6. Generation of cephalosporin courses in the analysis eTable 7. Change in cephalosporin use by generation (all courses) eTable 8. Ratios of ratios of odds ratios for changes in antibiotic use for patients with penicillin allergies eTable 9. Ratios of ratios of odds ratios for changes in oral antibiotic use for patients with penicillin allergies eTable 10. Ratios of ratios of odds ratios for changes in parenteral antibiotic use for patients with penicillin allergies eTable 11. Fraction of courses with a new allergy within 30 days eTable 12. Fraction of oral and parenteral courses with a new allergy within 30 days eTable 13. Odds ratios of new allergy within 30 days eTable 14. Crude odds ratios of antibiotic treatment failure within 30 days of a course of monotherapy eTable 15. Odds ratios of antibiotic treatment failure within 30 days of a course of monotherapy eTable 16. Crude and standardized mortality rates by penicillin allergy status and region eTable 17. Crude rate of hospital days per person year eTable 18. Crude rates of new infections per 1,000 person years eTable 19. Crude rates of new infections for patients with parenteral courses of antibiotics eTable 20. Ratios of ratios of odds ratios for change in cephalosporin use for patients with penicillin allergies from sensitivity analyses [file jamanetwopen-e218367-s001.pdf]

Supplementary Online Content

Macy E, McCormick TA, Adams JL, et al. Association between removal of a warning against cephalosporin use in patients with penicillin allergy and antibiotic prescribing. *JAMA Netw Open*. 2021;4(4):e218367. doi:10.1001/jamanetworkopen.2021.8367

eMethods. Additional methodology details

eTable 1. Antibiotic names and categories

eTable 2. Classification of cephalosporin antibiotics by generation

eTable 3. Definitions and modeling strategies for secondary outcomes

eTable 4. Descriptive statistics for courses in the analysis

eTable 5. Patterns of antibiotic use, by category, before and after warning was removed in the intervention site, by penicillin allergy status and region, for oral and parenteral courses separately

eTable 6. Generation of cephalosporin courses in the analysis

eTable 7. Change in cephalosporin use by generation (all courses)

eTable 8. Ratios of ratios of odds ratios for changes in antibiotic use for patients with penicillin allergies

eTable 9. Ratios of ratios of odds ratios for changes in oral antibiotic use for patients with penicillin allergies

eTable 10. Ratios of ratios of odds ratios for changes in parenteral antibiotic use for patients with penicillin allergies

eTable 11. Fraction of courses with a new allergy within 30 days

eTable 12. Fraction of oral and parenteral courses with a new allergy within 30 days

eTable 13. Odds ratios of new allergy within 30 days

eTable 14. Crude odds ratios of antibiotic treatment failure within 30 days of a course of monotherapy

eTable 15. Odds ratios of antibiotic treatment failure within 30 days of a course of monotherapy

eTable 16. Crude and standardized mortality rates by penicillin allergy status and region

eTable 17. Crude rate of hospital days per person year

eTable 18. Crude rates of new infections per 1,000 person years

eTable 19. Crude rates of new infections for patients with parenteral courses of antibiotics

eTable 20. Ratios of ratios of odds ratios for change in cephalosporin use for patients with penicillin allergies from sensitivity analyses

This supplementary material has been provided by the authors to give readers additional information about their work.

© 2021 Macy E et al. *JAMA Network Open*.

Association between removing a warning to avoid cephalosporin use in patients with penicillin allergy and antibiotic prescribing

Supplementary Appendix

Macy E, McCormick T, Adams J, et al.

Contents

eMethods. Additional Methodology Details 4
Study Population 4
Antibiotic Names and Categories 4
Study Definitions 4
Antibiotic Use 4
Anaphylaxis 4
New Antibiotic Allergy 4
Antibiotic Treatment Failure 4
All-Cause Mortality 5
Hospital Days 5
New Infections 5
Variables Used in Sensitivity Analyses 5
Statistical Analyses 5
Summary 5
Modeling of the Primary Outcome 6
Modeling of the Secondary Outcomes 6
Modeling of Outcomes for Patients with and Without Penicillin Allergies 6
Calculation of Confidence Intervals 6
Supplementary Tables 8
eTable 1. Antibiotic names and categories 8
eTable 2. Classification of cephalosporin antibiotics by generation 11
eTable 3. Definitions and modeling strategies for secondary outcomes 12
eTable 4. Descriptive statistics for courses in the analysis 14
eTable 5. Patterns of antibiotic use, by category, before and after warning was removed in the intervention site, by penicillin allergy status and region, for oral and parenteral courses separately 16
eTable 6. Generation of cephalosporin courses in the analysis 18
eTable 7. Change in cephalosporin use by generation (all courses) 19
eTable 8. Ratios of ratios of odds ratios for changes in antibiotic use for patients with penicillin allergies 20

© 2021 Macy E et al. *JAMA Network Open*.

eTable 9. Ratios of ratios of odds ratios for changes in oral antibiotic use for patients with penicillin allergies ... 21

eTable 10. Ratios of ratios of odds ratios for changes in parenteral antibiotic use for patients with penicillin allergies ... 22

eTable 11. Fraction of courses with a new allergy within 30 days ... 23

eTable 12. Fraction of oral and parenteral courses with a new allergy within 30 days ... 24

eTable 13. Odds ratios of new allergy within 30 days ... 25

eTable 14. Crude odds ratios of antibiotic treatment failure within 30 days of a course of monotherapy ... 26

eTable 15. Odds ratios of antibiotic treatment failure within 30 days of a course of monotherapy ... 27

eTable 16. Crude and standardized mortality rates by penicillin allergy status and region ... 28

eTable 17. Crude rate of hospital days per person year ... 29

eTable 18. Crude rates of new infections per 1,000 person years ... 30

eTable 19. Crude rates of new infections for patients with parenteral courses of antibiotics ... 31

© 2021 Macy E et al. *JAMA Network Open*.

eMethods. Additional Methodology Details

Study Population

Study inclusion and exclusion criteria were as follows:

- Inclusion:
  - Members enrolled in KPSC (the intervention site) or KPNC (the comparison site) who received any oral or parenteral antibiotic (dispensed/administered) listed in eTable 1 between January 1, 2017 and December 31, 2018
- Exclusion:
  - Antibiotics dispensed/administered during the wash out period (the 7 days before and 7 days after the December 20, 2017 change in the alert)
  - Antibiotics dispensed/administered outside of a membership period
  - Patients with missing birth date or gender
  - Patients with birth date after the date of the first prescription, administration, or dispense (which suggested an inaccurate birth date).
  - Patients with death date before the first administration or dispense (which suggested an inaccurate death date).

Antibiotic Names and Categories

The list of 144 antibiotic generic and class names used to identify relevant medication and allergy records is shown in eTable 1.

Study Definitions

Antibiotic Use

Periods of exposure to outpatient dispenses were calculated based on the dispense date and the days' supply, after adjusting sequential dispenses for stockpiled supply. Missing values of days' supply of outpatient medications were set to 1 day, and missing prescription dates were set to the date of the administration or dispense.

When two different antibiotics were administered in combination, they were considered to be two concurrent courses. For each course, we identified the date of the earliest prescription and the date of the earliest administration or dispense (the course start date). If any prescriptions in the course were associated with a diagnosis code, the earliest diagnosis code was used as the indication for the whole course.

For all courses, detailed route descriptions were categorized as either "oral" or "parenteral" to allow separate analyses by route of administration.

Anaphylaxis

We used ICD codes as described in the manuscript to identify potential cases of anaphylaxis. We included any outpatient coding of the relevant diagnoses on the same day as a parenteral course or within one day of an oral course of cephalosporin. For diagnoses that were coded during a hospitalization, it was not possible to determine from the secondary data the date on which the diagnosis occurred. Therefore, we included as potential cases all those coded during hospital or Emergency Department (ED) encounters that started within the same day as the start of a course of parenteral cephalosporin or within 1 day of a course of oral cephalosporin, or cases coded during a hospital stay that overlapped with the start of a course of a cephalosporin. Potential cases were confirmed by chart review, as described in the manuscript.

New Antibiotic Allergy

The calculations for new antibiotic allergies included courses for which the patient had at least 30 days of membership after the course start date, and included new allergy records and membership data through January 2019. The outcome was defined as a binary indicator for any new allergy records in the same antibiotic category during the 30-day follow-up window. New allergy records that appeared in the follow-up windows for multiple courses were assigned to the course(s) on the most recent date. If two courses in the same category started on the same date, the new allergy record was attributed to both (this accounted for approximately 2% of new allergy events).

Antibiotic Treatment Failure

The calculations for antibiotic treatment failure included courses of antibiotic monotherapy for which the patient had at least 30 days of membership in the analysis period after the course start date and included new courses of

© 2021 Macy E et al. *JAMA Network Open*.

antibiotics through December 2018. A course was considered to be monotherapy if it was the only course of antibiotics that the patient started on the date. For this calculation, cephalosporin generations 1, 2, and 3 (or higher) were treated as different categories of antibiotics (see eTable 2 for the mapping of cephalosporin generic names to generations). If a new course of antibiotics started in the follow-up window of multiple courses, it was considered treatment failure only for the most recent course; and the outcome was a binary indicator for the presence of any treatment failure events in the follow-up window.

All-Cause Mortality
The all-cause mortality calculation included person-time starting at the earliest course start date in the period (to avoid immortal time bias) and ended at the earlier of the period end or the death date. Age was calculated as of the first course start date in the period, and penicillin allergy status was assessed as of the day before.

Hospital Days
The hospital days per person-year calculation included all periods of membership during the analysis period as person-time and included all hospital encounters during these member periods. Membership periods and hospital stays were separated into days in the pre and post periods, and days with and without a penicillin allergy record.

New Infections
The calculations for each new infection type (*C. diff*, MRSA, and VRE) were carried out separately. Person-time was calculated from the first course date in the period until the earlier of the end of the period or the end of the membership period. The date associated with a lab result was the specimen collection date. The date associated with an outpatient or ED diagnosis was the encounter date. The date associated with a hospital diagnosis with an inpatient admission was the encounter end date, unless the diagnosis was flagged as being present on admission (in which case it was associated with the encounter start date). Diagnoses and lab results from encounters in the three months before the start of the analysis period were included to determine whether infections in January-March 2017 were new. Because a person could have recurrent infections, the outcome was defined as a binary indicator of zero vs. one or more infections during the period.

Variables Used in Sensitivity Analyses
For the sensitivity analyses, dates of the onset of the individual comorbidities in the Charlson index, as well as hypertension and hyperlipidemia, were extracted based on ICD-10 diagnosis codes from hospital and outpatient encounters. We extracted Diagnosis-Related Group (DRG) data for hospital encounters that overlapped with course time periods, and used the DRG and Major Diagnostic Category (MDC) codes to identify courses that occurred during hospitalizations that included surgeries or labor and delivery. Encounters in Hospital Ambulatory Surgery (HAS) were also counted as surgery encounters.

Statistical Analyses

Summary
A high-level summary of the outcome definitions and modeling strategies used in all main analyses is presented in eTable 3.

All models in the main analyses of the primary and secondary outcomes included the following variables:

- Main effects:
  - site (intervention or comparison)
  - period (pre or post)
  - penicillin allergy status
  - all two-way interactions of the above
  - the three-way interaction of the above (the coefficient on this interaction term is the logarithm of the RROR or RRRR of interest in each model)
- Covariates:
  - Sex
  - Age
  - race/ethnicity
  - the age-sex interaction

We adjusted for race/ethnicity in the multivariate analyses because of known differences between the patient populations of the two sites. Race and ethnicity are self-reported as part of routine data capture within the EHR. We

© 2021 Macy E et al. *JAMA Network Open*.

categorized the self-reported race/ethnicity values from the EHR into six categories (non-Hispanic American Indian/Alaska Native, non-Hispanic Asian/Pacific Islander, non-Hispanic Black, Hispanic, non-Hispanic White, and other). Other race/ethnicity includes those for whom race/ethnicity is unknown.

Modeling of the Primary Outcome

The primary outcome was change in antibiotic use, estimated at the course level. We used a generalized logistic regression model with penicillin as the reference level in the modeling of the primary outcome. The resulting estimate is an RROR representing the probability that a course of antibiotics will be cephalosporin.

Modeling of the Secondary Outcomes

We did not model probability of anaphylaxis because of small numbers (total of 68 potential cases, of which 9 were confirmed). To assess statistical significance of the anaphylaxis outcome (course level), we used Poisson regression on a person-level dataset with course count as an offset to calculate a chi-squared test with three degrees of freedom (based on two regions and two time periods).

We modeled all other secondary outcomes with binary logistic regression or Poisson regression, as described below. We also completed sensitivity analyses that are described below; the study findings did not change based on these models.

- New antibiotic allergies: we used binary logistic regression models to calculate the RROR for a new allergy in the same antibiotic category within 30 days of the start of a course of antibiotics, combining all antibiotic categories.
- Treatment failure: we used binary logistic regression models to calculate the RROR for the start of a course of antibiotics in a different category, within 30 days of the start of a course of monotherapy, combining all antibiotic categories.
- Mortality: crude mortality rates were directly standardized to the age, gender, and race/ethnicity distribution of the full intervention site pre period population. Separately, we used Poisson regression to estimate the change in all-cause mortality rates as a RRRR. As a sensitivity analysis, we fit a second model that included indicators for the presence of comorbidities (described above) as of the first course start in the period.
- Hospital Days: we used Poisson regression to estimate the change in hospital days as an RRRR. Because the same person could contribute both oral and parenteral courses to the analysis, this calculation could not be done separately for oral and parenteral courses. Instead, the model was also fit separately for patients who contributed parenteral courses to the analysis. As sensitivity analyses, we fit models that included indicators for the presence of comorbidities (described above) and with a more flexible negative binomial distribution.
- New Infections: we used logistic regression with a complementary log-log link to calculate the change in new infection rates as RRRRs (*C. diff*, MRSA, and VRE). The models were also fit separately for patients who contributed parenteral courses to the analysis. As a sensitivity analysis, we recalculated the new infection outcomes without censoring at the first membership gap.

Modeling of Outcomes for Patients with and Without Penicillin Allergies

We fit additional models of the secondary outcomes to estimate the odds ratio (or rate ratio) of the outcome for patients with penicillin allergies vs. patients without penicillin allergies. The independent variables in these models were the same as in the main outcome models except that they did not include interactions involving the penicillin allergy indicator. That is, the models included region, period, the region-period interaction, penicillin allergy status, sex, age, race/ethnicity, and the age-sex interaction.

Calculation of Confidence Intervals

For all model results reported with confidence intervals, Generalized Estimating Equations (GEEs) were used to fit models with a random 20% subset of the patients to estimate a multiplicative correction factor for standard errors related to patient-level correlations (since patients often contributed multiple courses in the analysis). This correction factor was used to increase the size of 95% confidence intervals for the full model results. Running these models on 100% of the data was computationally infeasible. The correction factors for most outcomes ranged from 1.0-1.2. The exception was the hospital days outcome, where the correction factor ranged from 4.3-4.4. We considered the possibility that correlations at a higher level of aggregation might also affect standard error and confidence interval calculations. To explore this, we fit alternative models that included fixed effects for medical office building area.

© 2021 Macy E et al. *JAMA Network Open*.

These geographic units correspond to the area around medical office buildings in the delivery system. The alternative models produced standard errors that were nearly the same as our main analyses.

© 2021 Macy E et al. *JAMA Network Open*.

Supplementary Tables
eTable 1. Antibiotic names and categories

Generic or class name	Category
AMDINOCILLIN	Penicillin
AMIKACIN	Other antibiotics
AMINOGLYCOSIDES	Other antibiotics
AMOXICILLIN	Penicillin
AMPICILLIN	Penicillin
AZITHROMYCIN	Macrolide
AZTREONAM	Other beta-lactam
CAPREOMYCIN	Other antibiotics
CARBAPENEMS	Other beta-lactam
CARBENICILLIN	Penicillin
CEFACLOR	Cephalosporin
CEFADROXIL	Cephalosporin
CEFAMANDOLE	Cephalosporin
CEFAZOLIN	Cephalosporin
CEFDINIR	Cephalosporin
CEFDITOREN	Cephalosporin
CEFEPIME	Cephalosporin
CEFIXIME	Cephalosporin
CEFMETAZOLE	Cephalosporin
CEFONICID	Cephalosporin
CEFOPERAZONE	Cephalosporin
CEFOTAXIME	Cephalosporin
CEFOTETAN	Cephalosporin
CEFOXITIN	Cephalosporin
CEFPODOXIME	Cephalosporin
CEFPROZIL	Cephalosporin
CEFTAROLINE	Cephalosporin
CEFTAZIDIME	Cephalosporin
CEFTIBUTEN	Cephalosporin
CEFTIZOXIME	Cephalosporin
CEFTOLOZANE	Cephalosporin
CEFTRIAXONE	Cephalosporin
CEFUROXIME	Cephalosporin
CEPHALEXIN	Cephalosporin
CEPHALOSPORINS	Cephalosporin
CEPHALOTHIN	Cephalosporin
CEPHAPIRIN	Cephalosporin
CEPHRADINE	Cephalosporin
CHLORAMPHENICOL	Other antibiotics
CHLORTETRACYCLINE	Tetracycline
CINOXACIN	Quinolone
CIPROFLOXACIN	Quinolone
CLARITHROMYCIN	Macrolide
CLINDAMYCIN	Clindamycin
CLOFAZIMINE	Other antibiotics
CLOXACILLIN	Penicillin
COLISTIMETHATE	Other antibiotics
COLISTIN	Other antibiotics

© 2021 Macy E et al. *JAMA Network Open*.

Generic or class name	Category
CO-TRIMOXAZOLE	Sulfonamide
CYCLOSERINE	Other antibiotics
DALBAVANCIN	Other antibiotics
DALFOPRISTIN	Other antibiotics
DAPSONE	Other antibiotics
DAPTOMYCIN	Other antibiotics
DELAFLOXACIN	Quinolone
DEMECLOCYCLINE	Tetracycline
DICLOXACILLIN	Penicillin
DIHYDROSTREPTOMYCIN	Other antibiotics
DIRITHROMYCIN	Macrolide
DORIPENEM	Other beta-lactam
DOXYCYCLINE	Tetracycline
ENOXACIN	Quinolone
ERTAPENEM	Other beta-lactam
ERYTHROMYCIN	Macrolide
ERYTHROMYCIN ETHYLSUCCINATE + SULFISOXAZOLE ACETYL	Macrolide and Sulfonamide
ETHAMBUTOL	Other antibiotics
ETHIONAMIDE	Other antibiotics
FIDAXOMICIN	Other antibiotics
FOSFOMYCIN	Other antibiotics
FURAZOLIDONE	Other antibiotics
GATIFLOXACIN	Quinolone
GEMIFLOXACIN	Quinolone
GENTAMICIN	Other antibiotics
GRAMICIDIN	Other antibiotics
GREPAFLOXACIN	Quinolone
IMIPENEM	Other beta-lactam
ISONIAZID	Other antibiotics
KANAMYCIN	Other antibiotics
LEVOFLOXACIN	Quinolone
LINCOMYCIN	Other antibiotics
LINEZOLID	Other antibiotics
LORACARBEF	Cephalosporin
MACROLIDES	Macrolide
MEROPENEM	Other beta-lactam
METHACYCLINE	Tetracycline
METHICILLIN	Penicillin
METRONIDAZOLE	Metronidazole
MEZLOCILLIN	Penicillin
MINOCYCLINE	Tetracycline
MONOBACTAMS	Other beta-lactam
MOXALACTAM	Cephalosporin
MOXIFLOXACIN	Quinolone
NAFCILLIN	Penicillin
NALIDIXIC ACID	Quinolone
NETILMICIN	Other antibiotics
NITROFURAN	Nitrofurantoin
NITROFURANTOIN	Nitrofurantoin
NORFLOXACIN	Quinolone
NOVOBIOCIN	Other antibiotics

© 2021 Macy E et al. *JAMA Network Open*.

Generic or class name	Category
OFLOXACIN	Quinolone
OLEANDOMYCIN	Macrolide
ORITAVANCIN	Other antibiotics
OXACILLIN	Penicillin
OXYQUINOLONE	Quinolone
OXYTETRACYCLINE	Tetracycline
PAROMOMYCIN	Other antibiotics
PEN G	Penicillin
PENICILLIN	Penicillin
PIPERACILLIN	Penicillin
POLYMYXIN	Other antibiotics
PYRAZINAMIDE	Other antibiotics
QUINOLONES	Quinolone
QUINUPRISTIN	Other antibiotics
RIFABUTIN	Other antibiotics
RIFAMPICIN	Other antibiotics
RIFAMPIN	Other antibiotics
RIFAMYCINS	Other antibiotics
RIFAPENTINE	Other antibiotics
RIFAXIMIN	Other antibiotics
ROLITETRACYCLINE	Tetracycline
SPARFLOXACIN	Quinolone
SPECTINOMYCIN	Other antibiotics
STREPTOMYCIN	Other antibiotics
SULFA (SULFONAMIDE ANTIBIOTICS)	Sulfonamide
SULFACYTINE	Sulfonamide
SULFADIAZINE	Sulfonamide
SULFADOXINE	Sulfonamide
SULFAMETHIZOLE	Sulfonamide
SULFAMETHOXAZOLE	Sulfonamide
SULFAPYRIDINE	Sulfonamide
SULFATHIAZOLE	Sulfonamide
SULFISOXAZOLE	Sulfonamide
TEDIZOLID	Other antibiotics
TELAVANCIN	Other antibiotics
TELITHROMYCIN	Macrolide
TETRACYCLINE	Tetracycline
TICARCILLIN	Penicillin
TIGECYCLINE	Tetracycline
TINIDAZOLE	Other antibiotics
TOBRAMYCIN	Other antibiotics
TRIMETHOPRIM	Sulfonamide
TRIMETREXATE	Other antibiotics
TROVAFLOXACIN MESYLATE	Quinolone
VANCOMYCIN	Vancomycin

© 2021 Macy E et al. *JAMA Network Open*.

eTable 2. Classification of cephalosporin antibiotics by generation

Generation	Generic
1	CEFADROXIL
1	CEFAZOLIN
1	CEPHALEXIN
2	CEFACLOR
2	CEFOTETAN
2	CEFOXITIN
2	CEFPROZIL
2	CEFUROXIME
3	CEFDINIR
3	CEFIXIME
3	CEFOTAXIME
3	CEFPODOXIME
3	CEFTAZIDIME
3	CEFTIBUTEN
3	CEFTRIAXONE
4	CEFEPIME
5	CEFTAROLINE
5	CEFTOLOZANE

© 2021 Macy E et al. *JAMA Network Open*.

eTable 3. Definitions and modeling strategies for secondary outcomes

	Outcome	Description	Model	Additional notes
**Course-level outcomes:**<br><br>**Models calculate ODDS RATIOS**	Change in Antibiotic Use	Probability that a course of antibiotics will be cephalosporin.	Multinomial logistic regression to calculate an RROR.	Primary outcome.
	Anaphylaxis	Counts of occurrence of anaphylaxis attributable to cephalosporin use, after a patient with a penicillin allergy uses a course of cephalosporin. Anaphylaxis must be coded on the same day as a parenteral course or within one day of the start of an oral course.	Not modeled due to small numbers.	This is the only outcome in the analysis that was limited to courses of cephalosporin among patients with penicillin allergies (this is the only outcome that involved manual chart review).
	New Antibiotic Allergy	Probability of a new allergy record in the same antibiotic category, within 30 days of the start of a course of antibiotics.	Binomial logistic regression to calculate an RROR.	Events are assigned to the most recent course only.
	Antibiotic Treatment Failure	Probability of the start of a course of antibiotics in a different category, within 30 days of the start of a course of monotherapy.	Binomial logistic regression to calculate an RROR.	Monotherapy is defined as being the only course of antibiotics that a patient starts on a date. Events are assigned to the most recent course only.
**Person-level outcomes:**<br><br>**Models calculate RATE RATIOS**	Mortality	All-cause mortality per person year.	Poisson regression to calculate an RRRR.	Person-time starts at the first course in the period (pre or post) and ends at death or the end of the period - no membership criterion.

© 2021 Macy E et al. *JAMA Network Open*.

	Hospital Days	Days in the hospital during an encounter that involves an inpatient admit, per person-year.	Poisson regression to calculate an RRRR.	Person-time includes all member days during the period (pre or post), including time before the first course.
	New Infection	Rate of one or more new C. diff, MRSA, or VRE infections per person-year.	Binomial logistic regression with a complementary log-log link, to calculate an RRRR.	Person time starts at the first course in the period (pre or post) and ends at the end of the period or the first membership gap, whichever happens first. An infection is new if there are no indications in the preceding 90 days (for C. diff) or 30 days (for MRSA or VRE).

© 2021 Macy E et al. *JAMA Network Open*.

eTable 4. Descriptive statistics for courses in the analysis

	Intervention	Comparison
Courses in the analysis	5,834,345	4,817,669
Courses in post period (%)	51.9	51.7
Courses that include any administrations (%)	20.1	21.9
Courses that include ED administrations (%)	3.9	6.6
Courses that include back-office administrations (%)	1.6	0.1
Courses that include any outpatient dispenses (%)	81.0	79.8
Courses that include a surgery encounter (%)	7.6	8.9
Courses that include a labor and delivery encounter (%)	1.3	1.6
Courses of penicillins (%)	26.4	26.1
Courses of cephalosporins (%)	25.7	23.0
Courses of other beta-lactams (%)	0.2	0.3
Courses of clindamycin (%)	3.7	3.7
Courses of macrolides (%)	13.1	13.8
Courses of metronidazole (%)	5.0	4.4
Courses of nitrofurantoin (%)	2.8	3.4
Courses of quinolones (%)	8.1	8.4
Courses of sulfonamides (%)	4.7	4.6
Courses of tetracyclines (%)	7.7	8.9
Courses of vancomycin (%)	1.4	1.7
Courses of any other antibiotics (%)	1.3	1.6
Oral courses (%)	82.7	81.2
Parenteral courses (%)	17.3	18.8
Any antibiotic allergy at start of course (%)	22.1	24.3
Allergies to more than 1 class (%)	6.0	6.9
Allergy to penicillins (%)	11.6	12.7
Allergy to cephalosporins (%)	2.6	2.6
Allergy to other beta-lactams (%)	0.0	0.1
Allergy to clindamycin (%)	0.9	0.8
Allergy to macrolides (%)	2.3	2.7
Allergy to metronidazole (%)	0.5	0.5
Allergy to nitrofurantoin (%)	0.8	0.7
Allergy to quinolones (%)	2.0	2.3
Allergy to sulfonamides (%)	8.0	9.3
Allergy to tetracyclines (%)	1.6	1.9
Allergy to vancomycin (%)	0.3	0.4

© 2021 Macy E et al. *JAMA Network Open.*

Allergy to any other antibiotic(s) (%)	0.3	0.4
Courses with any associated diagnosis (%)	75.6	64.7
First course dx category is Bone and joint (%)	0.6	0.4
First course dx category is Dental (%)	0.4	0.4
First course dx category is GI (%)	4.5	2.3
First course dx category is GU (%)	2.3	1.7
First course dx category is Infection (%)	0.1	0.1
First course dx category is Non-GI cancer (%)	0.3	0.1
First course dx category is Obstetric (%)	0.5	0.1
First course dx category is Pre-op (%)	1.2	0.3
First course dx category is Pulmonary (%)	3.6	3.2
First course dx category is Sepsis (%)	0.5	0.4
First course dx category is Sinus (%)	7.0	5.9
First course dx category is Skin infection (%)	8.4	7.8
First course dx category is URI (%)	15.8	13.2
First course dx category is UTI (%)	12.8	11.0
First course dx category is Other (%)	17.5	18.0
Courses with no associated diagnosis (%)	24.4	35.3

© 2021 Macy E et al. *JAMA Network Open*.

eTable 5. Patterns of antibiotic use, by category, before and after warning was removed in the intervention site, by penicillin allergy status and region, for oral and parenteral courses separately

		Intervention				Comparison			
	**Penicillin allergy flag status**	**No allergy**		**Allergy**		**No allergy**		**Allergy**	
	**Period**	**Pre**	**Post**	**Pre**	**Post**	**Pre**	**Post**	**Pre**	**Post**
**Oral courses**	Antibiotic courses	2,055,836	2,211,703	268,460	286,967	1,656,224	1,765,551	238,368	252,964
	Penicillin (%)	32.9	33.0	2.3	2.4	33.0	32.8	2.0	2.1
	Cephalosporin (%)	18.8	19.3	14.2	21.1	16.5	17.2	12.2	12.6
	Other beta-lactams (%)	0.0	0.0	0.0	0.0	0.0	0.0	0.0	0.0
	Clindamycin (%)	2.6	2.6	13.1	12.2	2.2	2.2	12.9	13.3
	Macrolides (%)	14.4	14.0	23.3	21.4	16.0	15.0	23.1	21.2
	Metronidazole (%)	4.7	4.8	4.9	4.9	3.9	4.4	4.7	4.7
	Nitrofurantoin (%)	3.0	3.4	5.3	4.8	3.8	4.2	5.2	5.7
	Quinolones (%)	8.7	8.1	13.1	11.2	8.3	7.8	14.1	13.6
	Sulfonamides (%)	5.5	5.2	9.0	7.1	5.5	5.1	8.8	8.3
	Tetracyclines (%)	8.4	8.6	13.5	13.6	9.4	10.0	15.3	16.8
	Vancomycin (%)	0.2	0.2	0.4	0.4	0.3	0.3	0.4	0.5
	Other antibiotics (%)	0.8	0.8	1.0	0.9	1.0	1.0	1.2	1.3
**Parenteral courses**	Antibiotic courses	424,699	467,404	56,396	62,880	373,049	408,816	59,342	63,355
	Penicillin (%)	13.1	13.0	1.3	1.4	15.4	15.6	1.9	1.9
	Cephalosporin (%)	59.7	60.1	35.5	54.3	55.5	55.7	27.6	30.3
	Other beta-lactams (%)	1.1	1.1	3.1	2.5	1.3	1.1	4.9	3.8
	Clindamycin (%)	2.2	2.2	16.5	7.5	2.2	2.1	18.5	17.7
	Macrolides (%)	3.3	3.4	3.5	3.9	2.7	2.9	2.2	2.2
	Metronidazole (%)	5.8	5.9	7.9	8.2	4.7	4.8	6.7	7.0
	Nitrofurantoin (%)	0.0	0.0	0.0	0.0	0.0	0.0	0.0	0.0
	Quinolones (%)	4.0	3.6	11.4	7.1	5.6	5.2	17.1	16.6

© 2021 Macy E et al. *JAMA Network Open*.

	Sulfonamides (%)	0.1	0.1	0.1	0.1	0.0	0.0	0.1	0.1
	Tetracyclines (%)	1.1	1.2	1.4	1.5	1.9	2.4	2.2	2.5
	Vancomycin (%)	6.1	6.2	13.4	9.7	6.8	6.7	13.1	12.5
	Other antibiotics (%)	3.5	3.3	6.0	3.8	3.8	3.6	5.6	5.3

The change in overall cephalosporin use was present in oral and parenteral courses separately, although the categories of antibiotics whose use decreased for patients in the intervention site with penicillin allergies differed slightly for the two routes. Both routes showed decreases in the use of clindamycin, macrolides, and quinolones. Oral sulfonamides, parenteral vancomycin, and parenteral "other antibiotics" also showed decreases.

© 2021 Macy E et al. *JAMA Network Open*.

eTable 6. Generation of cephalosporin courses in the analysis

Generation	Courses	% of cephalosporin courses
1	1,870,309	71.7%
2	135,925	5.2%
3	581,278	22.3%
4	21,795	0.8%
5	332	0.01%
All cephalosporins	2,609,639	100%

© 2021 Macy E et al. *JAMA Network Open*.

eTable 7. Change in cephalosporin use by generation (all courses)

	Intervention				Comparison			
**Penicillin allergy flag status**	**No allergy**		**Allergy**		**No allergy**		**Allergy**	
**Period**	**Pre**	**Post**	**Pre**	**Post**	**Pre**	**Post**	**Pre**	**Post**
Total courses	2,480,535	2,679,107	324,856	349,847	2,029,273	2,174,367	297,710	316,319
Fraction of courses that are:								
Cephalosporin (%)	25.8	26.4	17.9	27.0	23.7	24.4	15.3	16.2
1st generation (%)	18.4	18.8	9.6	16.2	18.0	18.6	8.6	9.1
2nd generation (%)	1.6	1.6	1.7	2.6	0.8	0.8	0.8	0.9
3rd generation (%)	5.6	5.8	6.3	7.8	4.7	5.0	5.6	5.9
4th generation (%)	0.2	0.2	0.4	0.5	0.1	0.1	0.3	0.3
5th generation (%)	0.003	0.003	0.003	0.005	0.003	0.003	0.006	0.003

© 2021 Macy E et al. *JAMA Network Open*.

eTable 8. Ratios of ratios of odds ratios for changes in antibiotic use for patients with penicillin allergies

	**Ratio of ratios of odds ratios**	
	**Crude (from Table 2)**	**From multinomial logistic regression (95% CI)**
Cephalosporin	1.47	1.47 (1.38-1.56)
Other beta-lactams	0.95	0.95 (0.83-1.09)
Clindamycin	0.83	0.83 (0.77-0.88)
Macrolides	0.99	0.98 (0.92-1.04)
Metronidazole	1.12	1.10 (1.03-1.18)
Nitrofurantoin	0.82	0.81 (0.75-0.87)
Quinolones	0.87	0.87 (0.81-0.92)
Sulfonamides	0.84	0.83 (0.78-0.89)
Tetracyclines	0.98	0.97 (0.91-1.04)
Vancomycin	0.82	0.82 (0.76-0.89)
Other antibiotics	0.83	0.83 (0.76-0.90)

© 2021 Macy E et al. *JAMA Network Open*.

eTable 9. Ratios of ratios of odds ratios for changes in oral antibiotic use for patients with penicillin allergies

	Ratio of ratios of odds ratios	
	Crude (from eTable 5)	From multinomial logistic regression (95% CI)
Cephalosporin	1.51	1.51 (1.41-1.61)
Other beta-lactams		
Clindamycin	0.94	0.93 (0.86-1.00)
Macrolides	1.00	0.99 (0.93-1.06)
Metronidazole	1.16	1.13 (1.05-1.23)
Nitrofurantoin	0.84	0.82 (0.76-0.89)
Quinolones	0.92	0.92 (0.85-0.98)
Sulfonamides	0.86	0.85 (0.79-0.91)
Tetracyclines	1.00	0.98 (0.92-1.05)
Vancomycin	1.05	1.05 (0.88-1.25)
Other antibiotics	0.99	0.98 (0.87-1.10)

© 2021 Macy E et al. *JAMA Network Open*.

eTable 10. Ratios of ratios of odds ratios for changes in parenteral antibiotic use for patients with penicillin allergies

	Ratio of ratios of odds ratios	
	**Crude (from eTable 5)**	**From multinomial logistic regression (95% CI)**
Cephalosporin	1.28	1.29 (1.10-1.51)
Other beta-lactams	0.83	0.84 (0.69-1.02)
Clindamycin	0.42	0.42 (0.36-0.50)
Macrolides	1.03	1.04 (0.86-1.27)
Metronidazole	0.92	0.92 (0.78-1.10)
Nitrofurantoin		
Quinolones	0.62	0.62 (0.53-0.74)
Sulfonamides	1.58	1.57 (0.70-3.49)
Tetracyclines	0.97	0.99 (0.79-1.23)
Vancomycin	0.69	0.70 (0.59-0.82)
Other antibiotics	0.63	0.63 (0.52-0.75)

© 2021 Macy E et al. *JAMA Network Open*.

eTable 11. Fraction of courses with a new allergy within 30 days

	Intervention				Comparison			
**Penicillin allergy flag status**	**No allergy**		**Allergy**		**No allergy**		**Allergy**	
**Period**	**Pre**	**Post**	**Pre**	**Post**	**Pre**	**Post**	**Pre**	**Post**
Penicillin (%)	0.64	0.61	1.00	1.02	0.85	0.78	1.29	1.05
Cephalosporin (%)	0.33	0.31	0.96	0.90	0.36	0.37	1.06	1.00
Other beta-lactams (%)	0.46	0.56	0.64	0.14	0.62	0.52	0.65	0.34
Clindamycin (%)	0.95	0.93	0.82	0.74	1.03	1.11	0.98	0.79
Macrolides (%)	0.21	0.17	0.34	0.33	0.21	0.16	0.35	0.33
Metronidazole (%)	0.34	0.35	0.50	0.59	0.40	0.37	0.52	0.60
Nitrofurantoin (%)	0.51	0.48	0.88	0.77	0.51	0.52	0.97	0.73
Quinolones (%)	0.55	0.57	0.94	0.96	0.72	0.78	1.16	1.09
Sulfonamides (%)	1.80	1.60	2.59	2.55	1.76	1.81	2.40	2.54
Tetracyclines (%)	0.41	0.39	0.76	0.67	0.46	0.42	0.85	0.81
Vancomycin (%)	0.74	0.76	0.78	0.92	0.78	0.83	0.80	0.99
Other antibiotics (%)	0.21	0.29	0.37	0.38	0.34	0.40	0.50	0.54
Total (%)	0.52	0.49	0.87	0.82	0.61	0.59	0.95	0.90

In total, 10,475,367 courses (98% of all courses in the analysis) had 30 days of follow-up and were included in the new allergy calculations. We identified 61,566 courses with a new allergy record in the same category within 30 days, corresponding to an overall new allergy rate of 0.6%. New allergy rates varied by antibiotic category and were more likely for patients that had a penicillin allergy, but generally decreased slightly in the post period. These general trends appeared in the data for all courses, as well as for oral and parenteral courses separately.

© 2021 Macy E et al. *JAMA Network Open*.

eTable 12. Fraction of oral and parenteral courses with a new allergy within 30 days

		Intervention				Comparison			
	**Penicillin allergy flag status**	**No allergy**		**Allergy**		**No allergy**		**Allergy**	
	**Period**	**Pre**	**Post**	**Pre**	**Post**	**Pre**	**Post**	**Pre**	**Post**
Oral courses	Penicillin (%)	0.66	0.63	1.01	1.05	0.89	0.81	1.40	1.07
	Cephalosporin (%)	0.40	0.37	1.16	1.10	0.46	0.48	1.27	1.19
	Other beta-lactams (%)	0.00	0.00	0.00	0.00	0.00	0.00	0.00	0.00
	Clindamycin (%)	1.02	0.98	0.97	0.77	1.14	1.21	1.20	0.96
	Macrolides (%)	0.21	0.17	0.34	0.32	0.21	0.16	0.35	0.34
	Metronidazole (%)	0.38	0.40	0.57	0.73	0.45	0.42	0.62	0.70
	Nitrofurantoin (%)	0.51	0.48	0.88	0.77	0.51	0.52	0.97	0.73
	Quinolones (%)	0.54	0.58	0.93	0.94	0.71	0.76	1.17	1.13
	Sulfonamides (%)	1.80	1.60	2.59	2.56	1.76	1.81	2.39	2.53
	Tetracyclines (%)	0.41	0.40	0.77	0.68	0.48	0.42	0.87	0.83
	Vancomycin (%)	0.24	0.22	0.23	0.52	0.23	0.15	0.11	0.74
	Other antibiotics (%)	0.29	0.43	0.60	0.53	0.48	0.55	0.71	0.74
	Total (%)	0.57	0.53	0.94	0.88	0.67	0.64	1.02	0.96
Parenteral courses	Penicillin (%)	0.35	0.33	0.89	0.74	0.44	0.45	0.80	0.95
	Cephalosporin (%)	0.22	0.20	0.55	0.53	0.22	0.24	0.67	0.66
	Other beta-lactams (%)	0.46	0.56	0.64	0.14	0.62	0.52	0.65	0.34
	Clindamycin (%)	0.52	0.65	0.26	0.50	0.56	0.65	0.35	0.28
	Macrolides (%)	0.19	0.20	0.33	0.54	0.32	0.20	0.34	0.08
	Metronidazole (%)	0.14	0.16	0.30	0.19	0.24	0.18	0.22	0.30
	Nitrofurantoin (%)	0.00	0.00	0.00	0.00	0.00	0.00	0.00	0.00
	Quinolones (%)	0.61	0.51	0.95	1.05	0.80	0.91	1.12	0.96
	Sulfonamides (%)	1.09	0.52	0.00	0.00	2.86	1.54	7.50	5.00
	Tetracyclines (%)	0.17	0.18	0.00	0.35	0.14	0.33	0.17	0.21
	Vancomycin (%)	0.84	0.86	0.86	0.99	0.89	0.96	0.89	1.03
	Other antibiotics (%)	0.12	0.14	0.19	0.22	0.17	0.21	0.32	0.35
	Total (%)	0.29	0.27	0.53	0.56	0.34	0.36	0.65	0.61

© 2021 Macy E et al. *JAMA Network Open*.

eTable 13. Odds ratios of new allergy within 30 days

	All courses		Oral courses		Parenteral courses	
	Crude odds ratio	Odds ratio from binomial logistic regression (95% CI)	Crude odds ratio	Odds ratio from binomial logistic regression (95% CI)	Crude odds ratio	Odds ratio from binomial logistic regression (95% CI)
Comparison, no penicillin allergy flag, post vs. pre	0.96	0.97 (0.94-1.00)	0.95	0.96 (0.93-0.99)	1.06	1.06 (0.97-1.15)
Intervention, no penicillin allergy flag, post vs. pre	0.94	0.95 (0.93-0.98)	0.94	0.95 (0.93-0.98)	0.96	0.96 (0.88-1.05)
Comparison, penicillin allergy flag, post vs. pre	0.95	0.95 (0.90-1.01)	0.95	0.95 (0.89-1.01)	0.94	0.94 (0.80-1.10)
Intervention, penicillin allergy flag, post vs. pre	0.94	0.95 (0.90-1.01)	0.94	0.94 (0.89-1.00)	1.05	1.05 (0.88-1.25)
Ratio ratios of odds ratios, Intervention/Comparison	1.02	1.02 (0.93-1.12)	1.00	1.00 (0.91-1.10)	1.23	1.23 (0.94-1.61)

The crude post/pre odds ratio for each combination of region and penicillin allergy status was smaller than 1 in most cases, consistent with the observation that the overall rate of new allergies decreased in the post period.

© 2021 Macy E et al. *JAMA Network Open*.

eTable 14. Crude odds ratios of antibiotic treatment failure within 30 days of a course of monotherapy

Route	Site	Penicillin allergy flag	Outcome odds		
			Pre	Post	Post/Pre
All Courses	Comparison	No	0.141	0.140	1.00
		Yes	0.175	0.176	1.00
	Intervention	No	0.152	0.151	0.99
		Yes	0.192	0.194	1.01
Oral	Comparison	No	0.122	0.121	0.99
		Yes	0.152	0.152	1.00
	Intervention	No	0.135	0.133	0.99
		Yes	0.170	0.172	1.01
Parenteral	Comparison	No	0.323	0.321	1.00
		Yes	0.406	0.408	1.00
	Intervention	No	0.345	0.342	0.99
		Yes	0.458	0.438	0.96

Of the courses with 30 days of follow-up, 8,177,655 (77% of all courses) were monotherapy and were included in the treatment failure calculations. We identified 1,072,038 courses with treatment failure events, corresponding to an overall rate of 13.1%. Treatment failure rates were higher for patients with penicillin allergies and after parenteral courses.

© 2021 Macy E et al. *JAMA Network Open*.

eTable 15. Odds ratios of antibiotic treatment failure within 30 days of a course of monotherapy

	All courses		Oral courses		Parenteral courses	
	Crude odds ratio	Odds ratio from binomial logistic regression (95% CI)	Crude odds ratio	Odds ratio from binomial logistic regression (95% CI)	Crude odds ratio	Odds ratio from binomial logistic regression (95% CI)
Comparison, no penicillin allergy flag, post vs. pre	1.00	0.99 (0.98-1.00)	0.99	0.99 (0.98-1.00)	1.00	0.99 (0.97-1.01)
Intervention, no penicillin allergy flag, post vs. pre	0.99	0.98 (0.98-0.99)	0.99	0.98 (0.97-0.99)	0.99	0.99 (0.97-1.01)
Comparison, penicillin allergy flag, post vs. pre	1.00	1.00 (0.98-1.02)	1.00	0.99 (0.97-1.01)	1.00	1.00 (0.96-1.05)
Intervention, penicillin allergy flag, post vs. pre	1.01	1.00 (0.99-1.02)	1.01	1.01 (0.99-1.03)	0.96	0.96 (0.92-1.01)
Ratio of odds ratios, Intervention/Comparison	1.02	1.02 (0.99-1.05)	1.02	1.02 (0.99-1.06)	0.96	0.96 (0.90-1.03)

© 2021 Macy E et al. *JAMA Network Open*.

eTable 16. Crude and standardized mortality rates by penicillin allergy status and region

Site	Penicillin allergy flag	Period	Mortality rate per 1000 person-years: Crude	Mortality rate per 1000 person-years: Standardized	Post/pre rate ratio
Comparison	no	pre	19.2	18.9	0.97
		post	18.8	18.3	
	yes	pre	29.5	19.5	0.97
		post	29.2	19.0	
Intervention	no	pre	15.3	16.2	0.98
		post	15.2	15.8	
	yes	pre	23.4	16.5	1.00
		post	24.4	16.6	

The crude overall mortality rate was 18.0 per 1,000 person-years. The rate decreased slightly in the post period in both regions, and was higher for patients in the comparison site and patients with penicillin allergies.

© 2021 Macy E et al. *JAMA Network Open*.

eTable 17. Crude rate of hospital days per person year

Site	Penicillin allergy flag	All patients			Patients with parenteral courses		
		Pre	Post	Post/Pre	Pre	Post	Post/Pre
Comparison	no	0.48	0.47	0.99	1.81	1.86	1.03
	yes	0.63	0.61	0.96	2.21	2.21	1.00
Intervention	no	0.46	0.45	0.99	1.65	1.69	1.03
	yes	0.57	0.57	1.00	1.96	2.05	1.04

The crude overall rate of hospital days was 0.48 days per person-year. The rates were higher for patients with penicillin allergies and for patients with any parenteral courses in the analysis.

© 2021 Macy E et al. *JAMA Network Open*.

eTable 18. Crude rates of new infections per 1,000 person years

	Site	Penicillin allergy flag	New infections		Rate per 1,000 person-years		
			Pre	Post	Pre	Post	Post/Pre
C. diff	Comparison	no	2,424	2,552	4.10	3.87	0.94
		yes	505	528	6.59	6.20	0.94
	Intervention	no	2,811	2,947	4.02	3.71	0.92
		yes	559	587	6.79	6.36	0.94
MRSA	Comparison	no	5,140	5,287	8.69	8.02	0.92
		yes	719	774	9.39	9.09	0.97
	Intervention	no	6,245	6,760	8.94	8.52	0.95
		yes	906	883	11.01	9.57	0.87
VRE	Comparison	no	466	384	0.79	0.58	0.74
		yes	124	101	1.62	1.19	0.73
	Intervention	no	503	574	0.72	0.72	1.01
		yes	141	129	1.71	1.40	0.82

We identified 12,913 new C. diff infections, 26,714 new MRSA infections, and 2,422 new VRE infections during the analysis period, which correspond to overall rates of 4.2 new C. diff infections, 8.7 new MRSA infections, and 0.79 new VRE infections per 1,000 person-years. The infection rates decreased slightly in the post period in both regions and were higher for patients with penicillin allergies.

© 2021 Macy E et al. *JAMA Network Open*.

eTable 19. Crude rates of new infections for patients with parenteral courses of antibiotics

	Site	Penicillin allergy flag	New infections		Rate per 1,000 person-years		
			Pre	Post	Pre	Post	Post/Pre
C. diff	Comparison	no	1,818	1,874	12.2	11.3	0.92
		yes	361	377	16.4	15.5	0.95
	Intervention	no	2,108	2,130	11.8	10.6	0.90
		yes	407	423	17.8	16.6	0.93
MRSA	Comparison	no	2,805	2,936	18.9	17.7	0.94
		yes	479	519	21.8	21.4	0.98
	Intervention	no	3,608	4,017	20.2	19.9	0.99
		yes	582	594	25.5	23.3	0.91
VRE	Comparison	no	422	356	2.84	2.15	0.76
		yes	115	95	5.22	3.92	0.75
	Intervention	no	472	547	2.64	2.72	1.03
		yes	131	115	5.74	4.51	0.79

© 2021 Macy E et al. *JAMA Network Open*.

eTable 20. Ratios of ratios of odds ratios for change in cephalosporin use for patients with penicillin allergies from sensitivity analyses

	**RROR from multinomial logistic regression (95% CI)**		
**Sensitivity analysis**	**All courses**	**Oral courses**	**Parenteral courses**
Result from model in main analysis (for comparison)	1.47 (1.38-1.56)	1.51 (1.41-1.61)	1.29 (1.10-1.51)
Model including only each person's first course	1.64 (1.47-1.84)	1.71 (1.53-1.92)	1.34 (0.99-1.81)
Model including only each person's last course	1.56 (1.40-1.75)	1.54 (1.38-1.73)	1.37 (1.03-1.81)
Model with allergy status as of course start date (rather than day before)	1.46 (1.37-1.55)	1.52 (1.41-1.63)	1.20 (1.03-1.41)
Model with additional covariates	1.58 (1.48-1.68)	1.59 (1.48-1.70)	1.36 (1.16-1.59)
Model with additional interactions	1.53 (1.44-1.63)	1.57 (1.46-1.68)	1.30 (1.10-1.52)

© 2021 Macy E et al. *JAMA Network Open*.
